# Alternative Practitioners Amuse the Patient, While Medics Cure the Disease

**DOI:** 10.3390/jcm7060137

**Published:** 2018-06-05

**Authors:** Edzard Ernst

**Affiliations:** Emeritus Professor, University of Exeter, UK; e.ernst@exeter.ac.uk

“The art of medicine consists in amusing the patient, while nature cures the disease”. During Voltaire’s time, his famous bon mot was largely correct. However, today, things are very different, and in the spirit of this Special Issue on a critical assessment of alternative medicine, I argue that it ought to be re-phrased as follows: “The art of alternative medicine consists in amusing the patient, while medics cure the disease”.

To illustrate this point, I shall use this editorial to tell the tale of a patient seeking care from a range of clinicians. The story is fictitious but nevertheless based on many real experiences of a similar nature.

Tom is in his mid-50s, happily married, mildly over-weight and under plenty of stress. In addition to having a demanding job, he has recently moved home. As a consequence of lots of heavy lifting, his body aches. He had previous episodes of back trouble and re-starts the exercises a physiotherapist once taught him. A few days later, the back-pain has improved, and most other pains have subsided as well. Yet a dull and nagging pain around his left shoulder and upper arm persists.

He is tempted to see his general practitioner (GP), but his wife is fiercely alternative. She was also the one who dissuaded Tom from taking statins for his high cholesterol and put him on garlic pills instead. Now she gives Tom a bottle of her Bach flower remedies, but after a week of taking it, Tom’s condition is unchanged. His wife therefore persuades him to consult alternative practitioners for his persistent ‘shoulder problem’. Thus, he sees a succession of her favourite clinicians.

The chiropractor examines Tom’s spine and diagnoses subluxations to be the root cause of his problem. Tom thus receives a series of spinal manipulations and feels a little improved each time. However, he is disappointed that the pain in the left shoulder and arm returns. His wife therefore makes another appointment for him.

The energy healer diagnoses a problem with Tom’s vital energy as the root cause of his persistent pain. Tom thus receives a series of healing sessions and feels a little improved each time. However, he is disappointed that the pain in the left shoulder and arm returns. His wife therefore makes another appointment for him.

The reflexologist examines Tom’s foot and diagnoses knots on the sole of his foot as causing energy blockages which are the root cause of his problem. Tom thus receives a series of most agreeable foot massages and feels a little improved each time. However, he is disappointed that the pain in the left shoulder and arm returns. His wife therefore makes another appointment for him.

The acupuncturist examines Tom’s pulse and tongue and diagnoses a chi deficiency to be the root cause of his problem. Tom thus receives a series of acupuncture treatments and feels a little improved each time. However, he is disappointed that the pain in the left shoulder and arm returns. His wife therefore makes another appointment for him.

The naturopath examines Tom and diagnoses some form of auto-intoxication as the root cause of his problem. Tom thus receives a full program of detox and feels a little improved each time. However, he is disappointed that the pain in the left shoulder and arm returns. His wife therefore makes another appointment for him.

The homeopath takes a long and detailed history and diagnoses a problem with Tom’s vital force to be the root cause of his pain. Tom thus receives a homeopathic remedy tailor-made for his needs and feels a little improved after taking it for a few days. However, he is disappointed that the pain in the left shoulder and arm returns. His wife therefore tries to make another appointment for him.

By this time, Tom had enough. Despite all these treatments, his pain has not substantially improved; on the contrary, he is increasingly feeling desperate and unwell. At the risk of a marital dispute, he consults his GP. The doctor looks up Tom’s history, asks a few questions, conducts a brief physical examination, and arranges for Tom to see a specialist. A cardiologist diagnoses Tom to suffer from coronary heart disease due to a stenosis in one of his coronary arteries. She explains that Tom’s dull pain in the left shoulder and arm is a rather typical symptom of this condition.

Tom has a stent put into the affected coronary artery, receives several medications for lowering his cholesterol and blood pressure, and is told to take up regular exercise, lose weight and make several other changes to his life-style. Tom’s wife is instructed in no uncertain terms to stop dissuading her husband from taking his prescribed medicines, and the couple are both sent to see a dietician who offers advice and recommends a course on healthy cooking. Nobody leaves any doubt that not following this complex (and holistic) package of treatments and advice would be a serious risk to Tom’s life.

It has taken a while, but finally Tom is pain-free. More importantly, his prognosis has dramatically improved. The team who now looks after him have no doubt that a major heart attack had been imminent, and Tom could easily have died had he continued to listen to the advice of multiple non-medically trained clinicians.

The root cause of his condition was misdiagnosed by all of them. In fact, the root cause was the atherosclerotic degeneration in his arteries. Even if the atherosclerotic process cannot be halted completely, it can be significantly slowed down such that Tom can live a full life.

My conclusion and advice based on this invented tale (and many real and stories of a very similar nature) is this:

- Alternative practitioners are often full of good intentions;
- They are often very good at pampering their patients;
- This may contribute to some perceived clinical improvements;
- In turn, this perceived benefit can motivate patients to continue their treatment despite residual symptoms;
- Alternative practitioner’s claims about ‘root causes’ and holistic care are usually pure nonsense;
- Their pampering may be agreeable, but it can undoubtedly cost lives.

We live in a time where alternative medicine is being seen as something harmless, and it is true that most treatments are not only safe but also quite pleasant. As we will discuss in this theme issue in some detail, this perception is perhaps not entirely correct and can even endanger the lives of patients.

